# Supplementary material for: Operando Li metal plating diagnostics via MHz band electromagnetics
Source: Nat Commun. 2023 Nov 10;14:7275. doi: 10.1038/s41467-023-43138-w (PMC10638420; doi:10.1038/s41467-023-43138-w)
Supplement: Supplementary file 1 — Supplementary Information [file 41467_2023_43138_MOESM1_ESM.pdf]

Supplementary Information for

Operando Li metal plating diagnostics via MHz band electromagnetics

Masanori Ishigaki[1], Keisuke Ishikawa[1], Tsukasa Usuki[1], Hiroki Kondo[1], Shogo Komagata[1] and Tsuyoshi Sasaki[1]

1) Secondary Battery Research Division, Toyota Central R&D Labs., INC. Japan

Supplementary Discussion 1: High-frequency current distribution on the boundary between two different conductivity materials

A cylindrical (radius = R, height = Z) coordinate system is used to analyse the high-frequency current density characteristics at the boundary of different conductive materials. Equation 1 is derived from Ohm's law: if the current density $\boldsymbol{i} = (i_z, i_r, i_\theta)$, electric field strength $\boldsymbol{E} = (E_z, E_r, E_\theta)$ and electrical conductivity $\sigma(z)$ are functions that fluctuate only in the Z direction,

$$\boldsymbol{i} = \boldsymbol{\sigma(z)E}, \tag{1}$$

$$\boldsymbol{\nabla} \times \boldsymbol{i} = \boldsymbol{\nabla} \times \sigma(z)\boldsymbol{E}. \tag{2}$$

Equation 2 can be described as Eqs. 3, 4 and 5 using Maxwell's equations $\boldsymbol{\nabla} \times \boldsymbol{E} = -\frac{\partial \boldsymbol{B}}{\partial \boldsymbol{t}} = -\mu\frac{\partial \boldsymbol{H}}{\partial \boldsymbol{t}}$, and Ampere's law $\boldsymbol{\nabla} \times \boldsymbol{H} = \boldsymbol{i}$, where $\boldsymbol{B} = (B_z, B_r, B_\theta)$ is the magnetic flux density, μ is the material permeability, $\boldsymbol{H} = \mu^{-1}\boldsymbol{B}$ is the magnetic field strength.

$$\boldsymbol{\nabla} \times \boldsymbol{i} = -\sigma(z)\mu\frac{\partial \boldsymbol{H}}{\partial t}, \tag{3}$$

$$\boldsymbol{\nabla} \times \boldsymbol{\nabla} \times \boldsymbol{i} = -\sigma(z)\mu\frac{\partial \boldsymbol{\nabla} \times \boldsymbol{H}}{\partial t}, \tag{4}$$

$$\boldsymbol{\nabla}(\boldsymbol{\nabla} \cdot \boldsymbol{i}) - \boldsymbol{\nabla}^2\boldsymbol{i} = -\sigma(z)\mu\frac{\partial \boldsymbol{i}}{\partial t}. \tag{5}$$

Current continuity ($\boldsymbol{\nabla} \cdot \boldsymbol{i} = \boldsymbol{0}$) simplifies Eq. 5 to Eqs. 6 and 7:

$$\boldsymbol{\nabla}^2\boldsymbol{i} = \sigma(z)\mu\frac{\partial \boldsymbol{i}}{\partial t}, \tag{6}$$

$$\frac{\partial^2 \boldsymbol{i}}{\partial r^2} + \frac{1}{r}\frac{\partial \boldsymbol{i}}{\partial r} + \frac{1}{r^2}\frac{\partial \boldsymbol{i}}{\partial \theta} + \frac{\partial^2 \boldsymbol{i}}{\partial z^2} = \sigma(z)\mu\frac{\partial \boldsymbol{i}}{\partial t}. \tag{7}$$

Finally, the behaviour of AC is described using Eq. 8 and $\boldsymbol{i} = \boldsymbol{I}e^{j\omega t}$:

$$\frac{\partial^2 \boldsymbol{I}}{\partial r^2} + \frac{1}{r}\frac{\partial \boldsymbol{I}}{\partial r} + \frac{1}{r^2}\frac{\partial \boldsymbol{I}}{\partial \theta} + \frac{\partial^2 \boldsymbol{I}}{\partial z^2} = j\sigma(z)\mu\omega\boldsymbol{I}. \tag{8}$$

Using Eq. 8, we estimate the current distribution on the σ interface. In the wide cylinder model, aspect $R >> Z$ allows $\frac{1}{r}\frac{\partial \boldsymbol{I}}{\partial r} \approx 0$, and symmetry allows $i_\theta = 0$.

If $\sigma(z)$ is a constant independent of z, then Eq. 8 can be represented as follows:

$$\frac{\partial^2 \boldsymbol{I}}{\partial r^2} = j\sigma(z)\mu\omega \boldsymbol{I}, \tag{9}$$

where

$$\boldsymbol{I} = Ae^{\left(\sqrt{\frac{\sigma(z)\mu\omega}{2}} \pm j\sqrt{\frac{\sigma(z)\mu\omega}{2}}\right)r}. \tag{10}$$

The well-known skin effect (Eq. 11) can be derived from Eq. 10.

$$\boldsymbol{I} = Ae^{\left(\sqrt{\frac{\sigma(z)\mu\omega}{2}}\right)r.} \tag{11}$$

In the battery model, $\sigma(z)$ in the layered materials is not constant and induces additional current distribution along the r axis. Based on the above equations, we observed a trend in $\boldsymbol{i_r}$ at each $\sigma(z)$ boundary. In each region, $\boldsymbol{i_z}$ attempts to distribute according to Eq. 12. In the boundary zone, $\boldsymbol{i_r}$ flows and varies continuously at the same time. The current continuity ($\boldsymbol{\nabla \cdot i = 0}$) changes the relation between $\boldsymbol{i_r}$ and $\boldsymbol{i_z}$:

$$\boldsymbol{\nabla \cdot i} = \frac{\partial \boldsymbol{i_z}}{\partial z} + \frac{1}{r}\frac{\partial r\boldsymbol{i_r}}{\partial r} + \frac{1}{r}\frac{\partial r\boldsymbol{i_\theta}}{\partial \theta} = 0$$

$$\frac{\partial \boldsymbol{i_z}}{\partial z} = -\frac{1}{r}\frac{\partial r\boldsymbol{i_r}}{\partial r}, \tag{12}$$

where i_θ is set to 0. When each region has $\sigma(z_1) = \sigma_1$ and $\sigma(z_2) = \sigma_2$, the approximated current $I_z = I_z(z,r)$ and $I_r = I_r(z,r)$ can be calculated in the neighborhood of the boundary $\Delta z = z_1 - z_2$,

$$\frac{\partial \boldsymbol{I_z}}{\partial z} = \frac{I_z(z_1,r) - I_z(z_2,r)}{z_1 - z_2}$$

$$= \frac{A(z_1)e^{\left(\sqrt{\frac{\sigma_1\mu\omega}{2}}\right)r} - A(z_2)e^{\left(\sqrt{\frac{\sigma_2\mu\omega}{2}}\right)r}}{\Delta z}$$

$$= -\frac{1}{r}\frac{\partial r I_r\left(\frac{z_1+z_2}{2}, r\right)}{\partial r}, \quad (13)$$

where $A(z)$ denotes area-specific constant amplitudes. The total current in the z direction $\int_0^{2\pi}\int_0^R I_z(z,r) r dr d\theta = constant$; hence, satisfy Eq. 15,

$$\int_0^{2\pi}\int_0^R A(z_1)e^{\left(\sqrt{\frac{\sigma_1\mu\omega}{2}}\right)r} r dr d\theta = \int_0^{2\pi}\int_0^R A(z_2)e^{\left(\sqrt{\frac{\sigma_2\mu\omega}{2}}\right)r} r dr d\theta \quad (14)$$

$$A(z_1)\left\{\frac{2}{\sigma_1\mu\omega}e^{\left(\sqrt{\frac{\sigma_1\mu\omega}{2}}\right)R}\left(\sqrt{\frac{\sigma_1\mu\omega}{2}}R - 1\right) + \frac{2}{\sigma_1\mu\omega}\right\}$$

$$= A(z_2)\left\{\frac{2}{\sigma_2\mu\omega}e^{\left(\sqrt{\frac{\sigma_2\mu\omega}{2}}\right)R}\left(\sqrt{\frac{\sigma_2\mu\omega}{2}}R - 1\right) + \frac{2}{\sigma_2\mu\omega}\right\}. \quad (15)$$

The trend of the boundary current $\boldsymbol{i_r}$ can be derived by reformulating Eqs. 13 and 15,

$$\frac{\partial r I_r\left(\frac{z_1+z_2}{2}, r\right)}{\partial r} = \frac{-A(z_1)e^{\left(\sqrt{\frac{\sigma_1\mu\omega}{2}}\right)r} + A(z_2)e^{\left(\sqrt{\frac{\sigma_2\mu\omega}{2}}\right)r}}{\Delta z} r \quad (16)$$

$$r I_r\left(\frac{z_1+z_2}{2}, r\right) = \int \frac{-A(z_1)e^{\left(\sqrt{\frac{\sigma_1\mu\omega}{2}}\right)r} + A(z_2)e^{\left(\sqrt{\frac{\sigma_2\mu\omega}{2}}\right)r}}{\Delta z} r dr$$

$$= \frac{-\frac{A(z_1)2}{\sigma_1\mu\omega}e^{\left(\sqrt{\frac{\sigma_1\mu\omega}{2}}\right)r}\left(\sqrt{\frac{\sigma_1\mu\omega}{2}}r - 1\right) + \frac{A(z_2)2}{\sigma_2\mu\omega}e^{\left(\sqrt{\frac{\sigma_2\mu\omega}{2}}\right)r}\left(\sqrt{\frac{\sigma_2\mu\omega}{2}}r - 1\right)}{\Delta z}$$

$$I_r\left(\frac{z_1+z_2}{2}, r\right) = \frac{-\frac{A(z_1)2}{\sigma_1\mu\omega}e^{\left(\sqrt{\frac{\sigma_1\mu\omega}{2}}\right)r}\left(\sqrt{\frac{\sigma_1\mu\omega}{2}}r - 1\right) + \frac{A(z_2)2}{\sigma_2\mu\omega}e^{\left(\sqrt{\frac{\sigma_2\mu\omega}{2}}\right)r}\left(\sqrt{\frac{\sigma_2\mu\omega}{2}}r - 1\right)}{r\Delta z}. \quad (17)$$

When σ_1 is the conductivity of the active material and σ_2 is the conductivity of the electrolytic solution, we can estimate the vertical current of the electrolytic solution layer as $I_z(z_2, r) = constant = B$ with $\sigma_2\mu\omega \ll \sigma_1\mu\omega$. Then, Eq. 17 is simplified as follows:

$$I_r\left(\frac{z_1+z_2}{2}, r\right) = \frac{-\frac{A(z_1)2}{\sigma_1\mu\omega} e^{\left(\sqrt{\frac{\sigma_1\mu\omega}{2}}\right)r}\left(\sqrt{\frac{\sigma_1\mu\omega}{2}}r - 1\right) + \frac{1}{2}Br^2}{r\Delta z}, \qquad (18)$$

where $A(z_1)$ can be described by B in Eq. 15:

$$A(z_1) = \frac{BR^2\frac{\sigma_1\mu\omega}{2}}{2\left(e^{\left(\sqrt{\frac{\sigma_1\mu\omega}{2}}\right)R}\left(\sqrt{\frac{\sigma_1\mu\omega}{2}}R - 1\right) + 1\right)}. \qquad (19)$$

Equation 18 summarizes the trend of the current distribution in the surface direction (r) on the higher conductivity surface of different conductivity materials. Moreover, this current is increased monotonically by σ_1. Consequently, the Li-metal plating that increases the digits of σ_1 significantly changes the H_z of a battery.

Supplementary Table 1: Specifications of the tested batteries

Model name	NCR18650b	INR18650 M26	IFR18650EC-1.5Ah
Sample name	NCA	NCM	LFP
Battery type	18650		
Anode material	Graphite		
Cathode material	$LiNi_{0.8}Co_{0.15}Al_{0.05}O_2$	$LiNi_{0.49}Co_{0.2}Mn_{0.31}O_2$	$LiFePO_4$
Nominal voltage	3.6 V	3.65 V	3.2 V
Cutoff voltage	2.5–4.2 V	2.75–4.2 V	2–3.65 V
Nominal capacity	3350 mAh	2600 mAh	1500 mAh
Weight	48.5 g	44.0 g	42.0 g
Standard charge current	1625 mA	1250 mA	300 mA
Max charge current	1675 mA	2500 mA	3000 mA

Supplementary Table 2: Degradation conditions of the 18650 batteries

**Sample name**	**NCA**		**NCM**		**LFP**	
Degradation name	Excessive rapid charge cycle	High-temperature cycle	Excessive rapid charge cycle	Excessive rapid charge cycle with sensor	Excessive rapid charge cycle	High-temperature cycle
Temperature	20 ˚C	60 ˚C	20˚C	20˚C	20˚C	60˚C
CC charge	6700 mA (2 C) Cutoff 4.2 V	1675 mA (0.5 C) Cutoff 4.2 V	5200 mA (2 C) Cutoff 4.2 V	5200 mA (2 C) Cutoff 4.3 V	6000 mA (4 C) Cutoff 3.9 V	750 mA (0.5 C) Cutoff 3.65 V
CC discharge	335 mA (0.1 C) Cutoff 2.5 V	1675 mA (0.5 C) Cutoff 2.5 V	260 mA (0.1 C) Cutoff 2.75 V	1300 mA (0.5 C) Cutoff 2.85 V	150 mA (0.1 C) Cutoff 2.0 V	750 mA (0.5 C) Cutoff 2.0 V
CV discharge	2.5 V 3 h	-	2.75 V 3 h	2.85 V 3 h	2.0 V 3 h	-
Rest time after charge/discharge	10 min	10 min	10 min	10 min	10 min	10 min
Cycle number	12–63	80–575	5–90	31	6–30	20–300

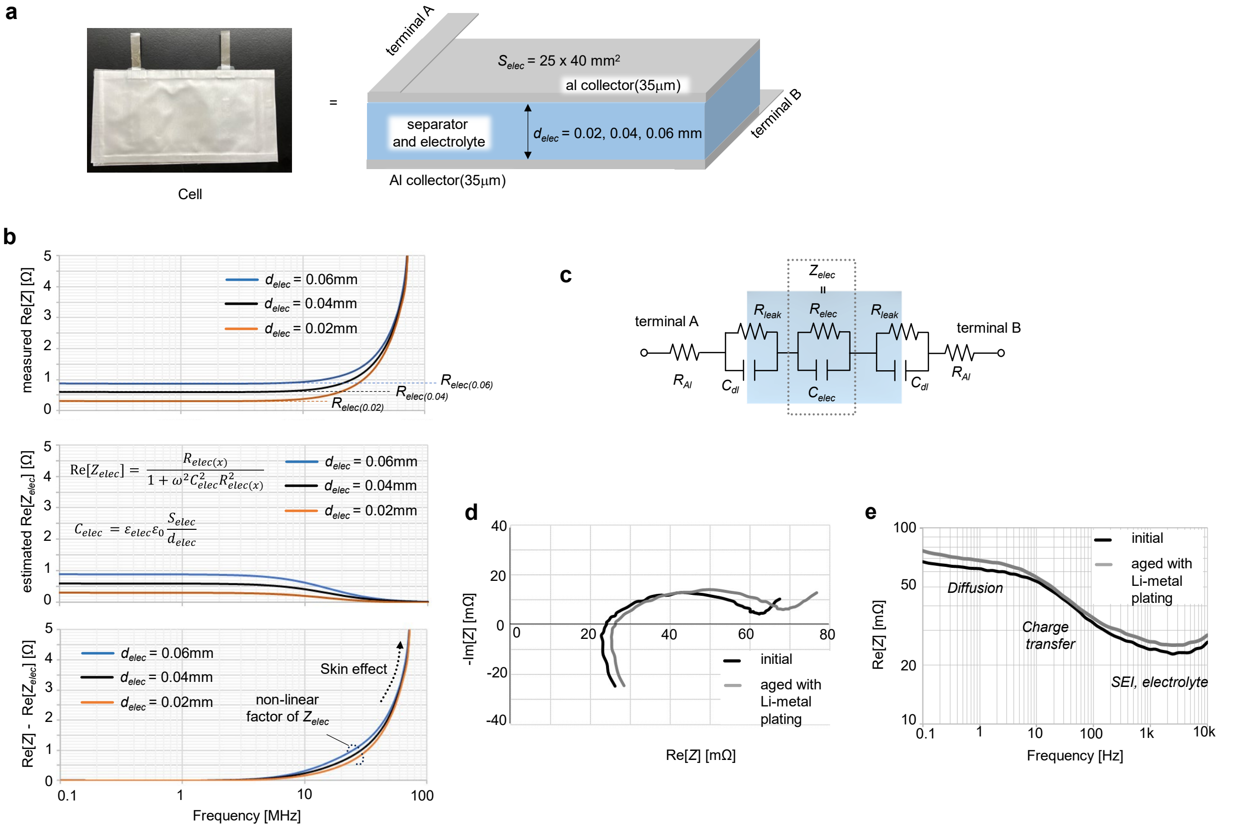

Supplementary Fig. 1 | Verification of the ECM for EIS. a-c The ECM of electrolyte Z_{elec} at high frequency. **a** A cell configuration for verifying the high-frequency response of the Z_{elec}. The cell is designed as an electric double-layer capacitor that uses the same electrolyte and separator as the laminate cell shown in the Supplementary Fig. 3. The thickness of the electrolyte is changed by stacking the separator. **b,c** Frequency characteristics of the measured real part of the impedance Re[Z] and estimated equivalent circuit of Z_{elec}. R_{elec} represents the electrolyte resistance, and C_{elec} represents the geometrical stray capacitance between the collector plates. The R_{elec} is proportional to the thickness of the electrolyte up to 4 MHz. However, Re[Z] converges to the same value from 70 MHz with increasing value by the skin effect and the proximity effect. This behaviour can be modelled by the RC model shown in (c), which uses the conductivity and relative dielectric constant as σ_{elec}= 0.01 [S/m] and $\varepsilon_{\mathrm{elec}}$ = 81, respectively, which are used in other simulations, such as the Supplementary Fig. 2. The estimated RC model has a cut-off of approximately 10 MHz, and Re[Z_{elec}] can converge to zero by increasing the frequency. In addition, R_{elec} can be sufficiently small by increasing the cross-sectional area of S_{elec} in a large-capacity battery. Therefore, the high-frequency measurement can deal with ionic degradation as negligible, and even Z_{elec} might change its resistance by degradation. **d,e** Example of a low-frequency EIS result in the 18650-type battery ID [#Z] shown in Fig. **4**, which has a Li-metal plate by degradation. **d** The Cole-Cole plot from 0.1Hz to 10 kHz. **e** Re[Z] vs. frequency. The resistance is broadly increased by Li-metal plating mixed with other degradation factors.

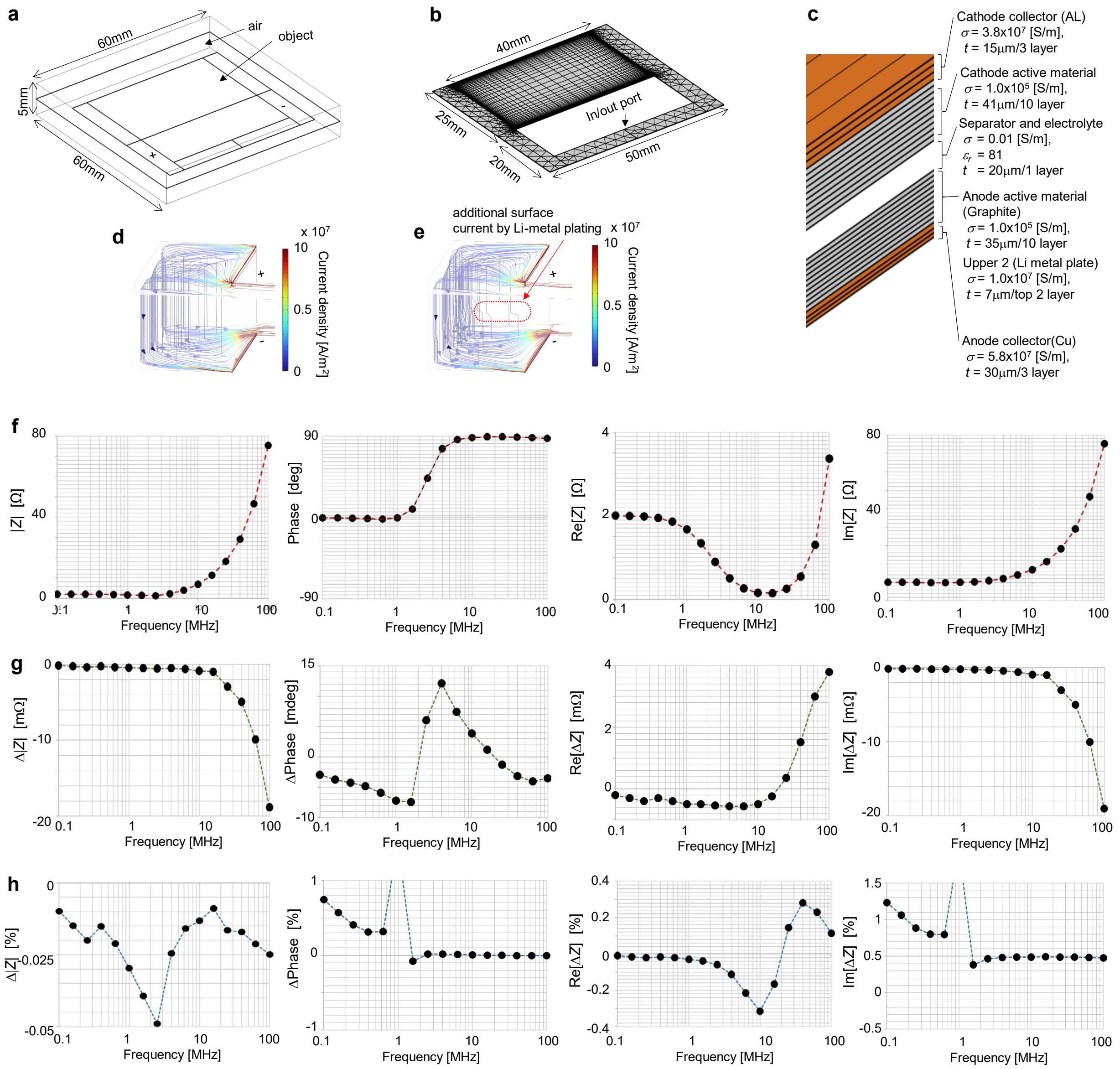

Supplementary Fig. 2 | Laminate cell modelling and visualization of high-frequency electromagnetic behaviour by computational analysis. a Overall negative-facing cell model in a high-frequency multiphysics simulation (COMSOL6.0). The cell size is referenced in the laminated pouch cell used in the experimental result shown in the Supplementary Fig. 3. **b** Mesh design. A squared mesh was manipulated as the gradationed size in the edges for monitoring the high-frequency current concentration. **c** Design of the lamination slices and parameters. The graphite layer was sliced into 10 layers, and special conductivity can be applied to the top two graphite layers to emulate Li-metal plating. **d** Simulation result of the overall current flow at 10 MHz without Li-metal plating. The current spread in the top and bottom collector layers and down straight in the other layers. **e** Simulation result of the overall current flow at 10 MHz with the Li-metal layer. There is an additional surface current in the middle of the battery, where the conductivity changes from that of graphite to that of Li metal. **f,g,h** Overall simulation results. ΔZ is calculated by the difference between (d) and (e).

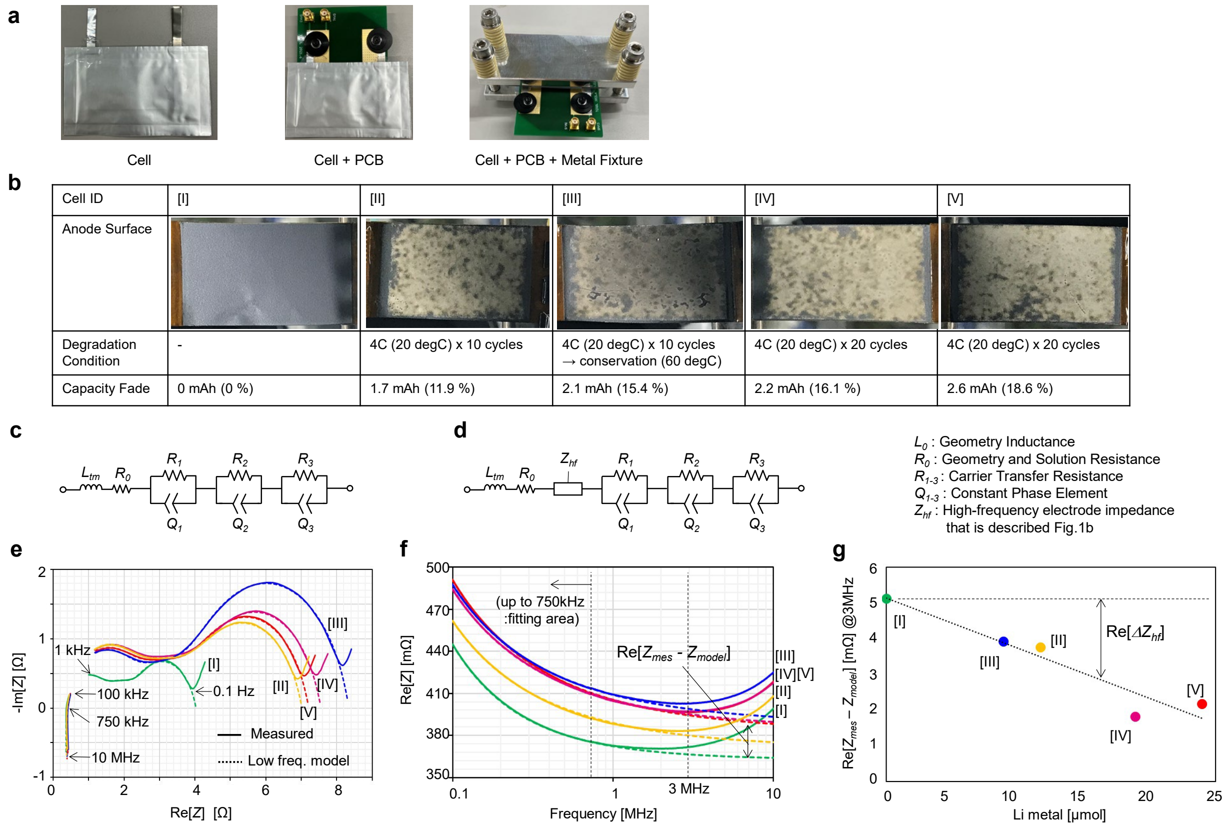

Supplementary Fig. 3 | Experimental verification of the negative correlation between Li metal and high-frequency impedance (analytical inspection). a Photographs of the laminate-type pouch cell. The cells were connected to the PCB with SMA connectors. **b** Table summarising the results of the degradation tests conducted on the laminate-type pouch cells, including images of the anode, degradation conditions and capacity loss of the Li-deposited cells. [I] Initial state of the battery used as a reference. Li metal is deposited in [II][IV][V] batteries via cycle tests. Following a cycle test, [III] is exposed to a storage test at a high temperature. At high temperatures, some of the precipitated Li metal transformed into SEI, resulting in the formation of dark brown precipitates. **c** Conventional ECM valid for low-frequency[10]. **d** Proposed ECM applicable to the high-frequency region. The presence of high-frequency impedance Z_{hf}, shown as impedance behaviour in the MHz band, including Li-metal plating, deviates from the conventional ECM. **e** Cole-Cole plot of the measured impedance curves (Z_{mes}) and fitting curves (Z_{model}) by the conventional ECM. These curves were fitted in the range of 100 mHz to 750 kHz and showed good agreement with the measured values at low frequencies, regardless of the degradation condition. **f** Comparison of the measured and fitted values of the real component of the impedance at high frequency. The measured and fitted values were consistent up to 1 MHz but from 2MHz to 3 MHz. The measured values started to increase, whereas the fitted values monotonically decreased. This difference corresponded to Z_{hf}. **g** Relationship between the Li metal and Re[$Z_{mes} - Z_{model}$]. The negative correlation between the volume of the Li metal and Re[$Z_{mes} - Z_{model}$] can be confirmed. In the electromagnetic simulation, the distinction between [I] and [II]–[V] is equivalent to the Re[ΔZ] in Fig. **3**.

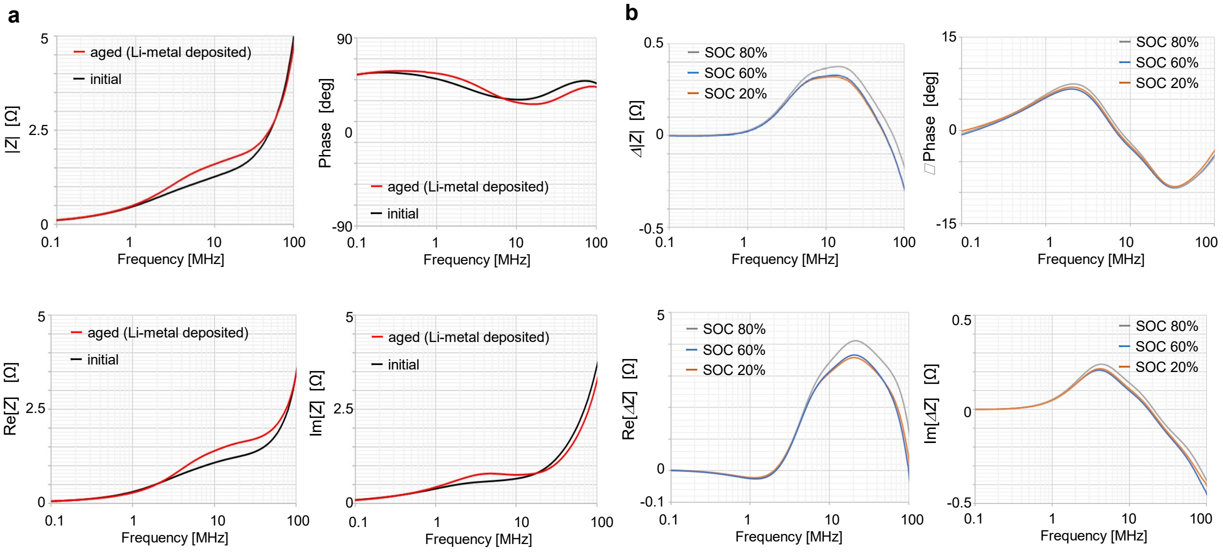

Supplementary Fig. 4 | Overall high-frequency measurement result of the 18650-type battery used in Fig. 4 (1500 mAh, LFP). a Frequency vs. impedance from 0.1MHz to 100 MHz. Impedance $|Z|$ and Re[Z] are proportional to the frequency. Compared to the laminated pouch cell shown in Supplementary Fig. 3, an 18650-type battery behaves as an inductor in this frequency range. In the 10 MHz frequency band, both the real and imaginary components have a convex profile. This local behaviour suggests a second potential for this high-frequency diagnosis that directly monitors the status of health (SOH), as summarised in the Supplementary Fig. 5. **b** ΔZ vs. frequency with dependency against the state of charge (SOC). Re[ΔZ] is stable against the SOC status in the MHz range used for Li-metal detection. This feature is important for field use since it does not require battery conditioning equipment such as precise charger systems for SOC adjustment.

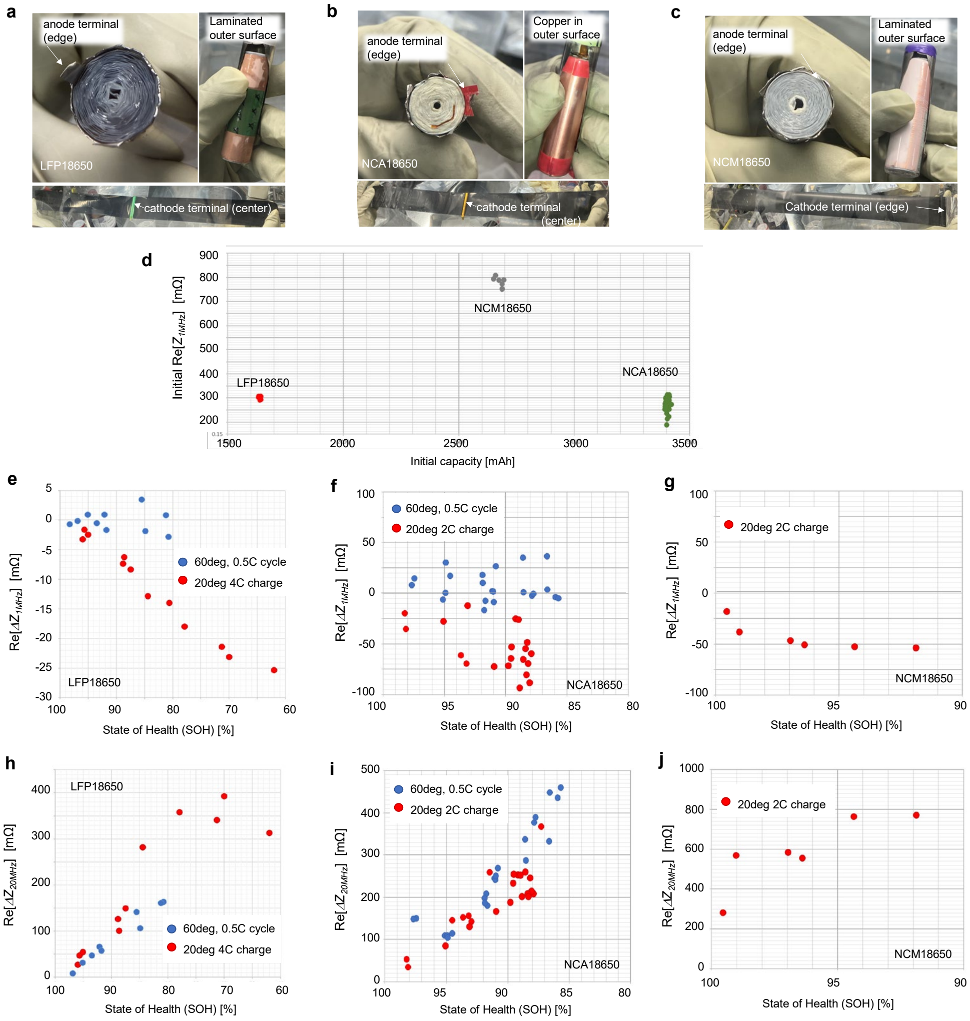

Supplementary Fig. 5 | Further experimental results for 18650-type batteries. a-d Geometrical features and initial characteristics of the evaluated 18650-type batteries. Battery (a) is LFP/1500 mAh with Re[Z_{1MHz}] = 300 mΩ as the initial value. Battery (b) is NCA/3350 mAh that has Re[Z_{1MHz}] = 290 mΩ as the initial value. Battery (c) is NCM/2500 mAh and has Re[Z_{1MHz}] = 780 mΩ as the initial value. Battery cells (a) and (b) have the cathode terminal in the center of the aluminium collector. The battery cell (c) has the cathode terminal on the edge of the aluminium collector. Only battery cell (b) has an anode collector (copper) in the outer surface, whereas the others have dielectric films between its housing. In the battery shown in (c), only excessive rapid charge degradation results are measured due to a lack of sample cells. **e-g** Measured impedance change at 1 MHz. The results show that our method is not affected by the material but is affected by the structural features. **e,h** The results of the LFP-type (Repost of Fig. **4**) and the NCM-type. Since battery (c) has the longest terminal-to-terminal edge, the negative collation between SOH and Re[ΔZ_{1MHz}] in (f) can be observed to be stronger than that in other batteries. **f** The result of the NCA-type is scattered compared to the LFP-type (d) or NCM-type (f). The reason for this scattering is assumed to be geometrical noise. The unstable outer contact clearly affects the high-frequency impedance by interfering with the anode collector's current flow. **h-j** Measured impedance change at 20 MHz in each battery. In contrast to the 1 MHz results, both blue and red dots are blended and aligned along the proportionate lines against the SOH. Although a scientific review is needed, it might be applied as a technique for rapid capacity estimation.

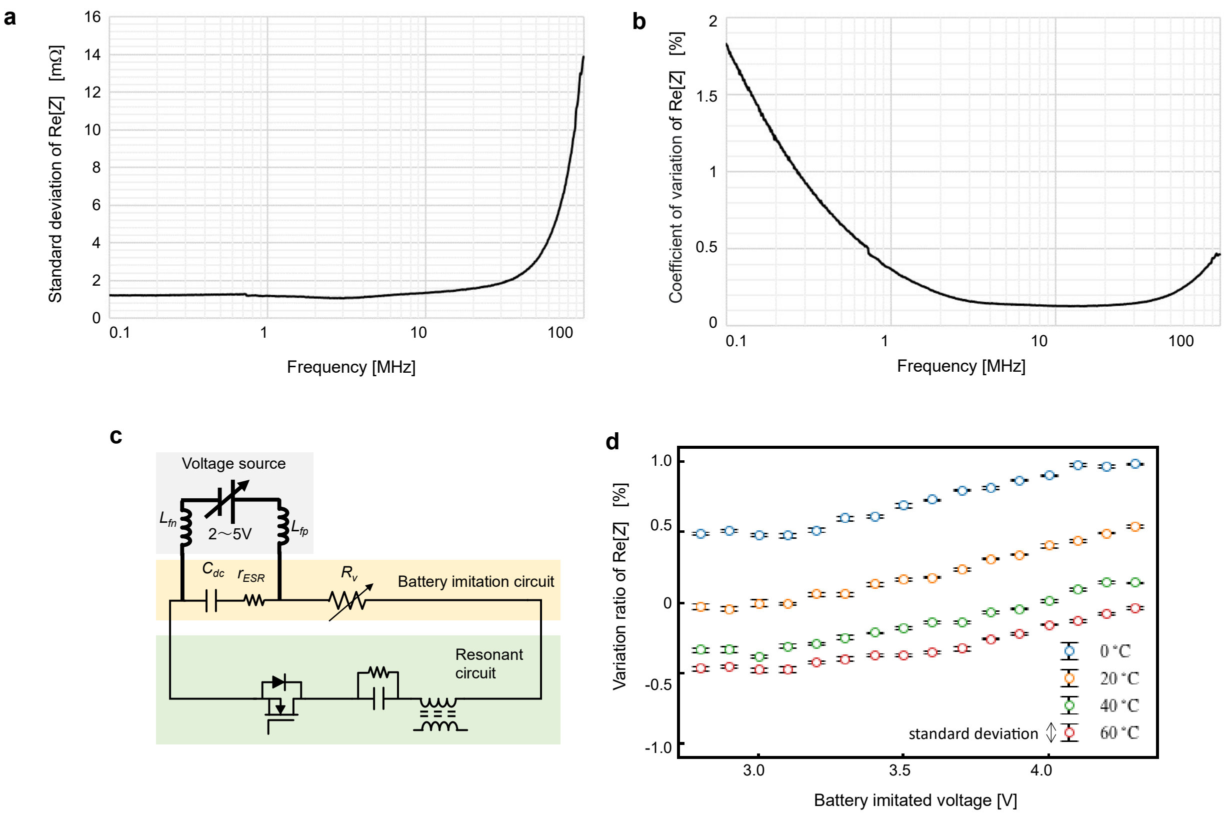

Supplementary Fig. 6 | Accuracy verifications of the high-frequency impedance measurements. a,b Characteristics of the network analyser using a shunt-through measurement method. The standard deviation and coefficient of variation are measured by repeatedly removing and reinserting the battery socket five times. The test battery is the LFP-18650 used in Fig. **4**. The battery condition is not degraded, the SOC is set to 0%, and the battery temperature is controlled at 20 °C by a constant-temperature chamber. **a** Standard deviation of measured Re[Z] vs. frequency. The standard deviation of the real part of the impedance is less than 1.61 mΩ in the range of 100 kHz to 20 MHz. The average real part of the impedance is 329 mΩ at 1 MHz. **b** The coefficient of variation of Re[Z] vs. frequency. The coefficient of variation is less than 0.37 % in the range of 1 MHz to 20 MHz. The results are sufficiently accurate to measure the change in Re[Z]. **c,d** Characteristics of the operando sensor shown in Fig. **5**. **c** Test configuration. A series connected to C_{dc} and r_{ESR} and R_v imitates a battery cell, where C_{dc} is able to change its voltage via a voltage source for emulating the battery voltage. The filter characteristics of L_{fp}, L_{fn} (10 µH in each, ferrite inductor) and C_{dc} (50 µF, ceramic capacitor) constrain the path of high-frequency currents, not to through the voltage source. **d** Evaluation of voltage and temperature dependency of the prototype sensor with error ratio. The sensor characteristics are evaluated by using an imitated battery that has C_{dc}=100 µF and r_{ESR}+ R_v=300 mΩ. The real part of the impedance Re[Z] at 3 V and 20 °C is set as the reference value, and a variation ratio from the reference is calculated at each point. The error bar (Standard deviation) in each point includes the white noise of the sensor. At each point, the error bar is less than 0.1%. The variation ratio against voltage (from 2.8 to 4.3 V) and temperature (from 0 to 60 °C) is less than ±1%. These results indicate that the sensor has enough accuracy for tracking the impedance change in Fig. **5**, which varies the value of Re[Z] by more than 5 mΩ (1.6% of 300 mΩ) by Li-metal plating.
